# An optimized workflow for CRISPR-Cas9 deletion of surface and intracellular factors in primary human T lymphocytes

**DOI:** 10.1371/journal.pone.0247232

**Published:** 2021-02-18

**Authors:** Cristina Leoni, Niccolò Bianchi, Lucia Vincenzetti, Silvia Monticelli

**Affiliations:** 1 Institute for Research in Biomedicine (IRB), Università della Svizzera italiana (USI), Bellinzona, Switzerland; 2 Graduate School for Cellular and Biomedical Sciences, University of Bern, Bern, Switzerland; West China Hospital, Sichuan University, CHINA

## Abstract

The appropriate regulation of T lymphocyte functions is key to achieve protective immune responses, while at the same time limiting the risks of tissue damage and chronic inflammation. Deciphering the mechanisms underpinning T cell responses in humans may therefore be beneficial for a range of infectious and chronic diseases. Recently, the development of methods based on CRISPR-Cas9 gene-editing has greatly expanded the available tool-box for the mechanistic studies of primary human T cell responses. While the deletion of a surface protein has become a relatively straightforward task, as long as an antibody for detection is available, the identification and selection of cells lacking an intracellular protein, a non-coding RNA or a protein for which no antibody is available, remain more problematic. Here, we discuss the options currently available to scientists interested in performing gene-editing in primary human T lymphocytes and we describe the optimization of a workflow for the screening and analysis of lymphocytes following gene-editing with CRISPR-Cas9 based on T cell cloning and T7 endonuclease I cleavage assay.

## Introduction

The availability of efficient and reliable gene editing tools is important not only for basic discoveries, leading to a better understanding of the regulatory networks of immune responses, but potentially also for therapy. For instance, engineering the genome of T lymphocytes holds great promise for cancer immunotherapy, and emerging therapies can be envisioned also for at least some infectious and autoimmune diseases. Gene-editing approaches exploiting the CRISPR-Cas9 technology have quickly become the tools of choice to generate deletions and gene modifications in a wide range of cell types and species, including primary human T lymphocytes [1–7]. Cas9 is a programmable DNA endonuclease that can be directed to specific loci in the genome by its associated guide RNA (gRNA). Cas9 directly targets the genomic DNA (gDNA) for cleavage, leading to double-strand breaks that, when imperfectly repaired, result in insertions or deletions (indels) in the gene of interest. Such disruption of the coding sequence can result in protein ablation. The advantages of this method are first and foremost linked to the permanent alteration of the cellular DNA, allowing long-term studies of the functionality of any given gene. Moreover, off-target effects of Cas9 appear to be limited [8, 9], and the system is sufficiently versatile to allow the insertion of reporter genes such as GFP in a desired genomic location [2, 10], thereby providing a variety of approaches for the interrogation of complex biological questions. In comparison, other tools for gene perturbation, such as gene knockdown by RNA interference (RNAi) may suffer of transient efficacy, reduced ability to eliminate long-lived proteins and relatively high off-target effects [11]. Although gene-editing with CRISPR-Cas9 provides tremendous potential to address a wide range of questions, at the moment the method is also not fully devoid of shortcomings, at least when used as a discovery tool for the mechanistic analysis of primary human T lymphocytes. Indeed, complete ablation of a given protein after gene-editing is normally achieved only in a subset of cells, and the current protocols often require selection of the effectively deleted population using antibodies, prior to functional analyses. While this is relatively easy to accomplish for most labs in the case of surface proteins through the use of a cell sorter, in the case of intracellular proteins, the selection of deleted cells would require cell permeabilization for intracellular staining, and is therefore incompatible with the analysis of live cells. Attempting the deletion of non-coding RNAs such as microRNAs would also be impeded by similar problems of cell selection. Moreover, the use of other antibody-based systems such as Western blots is often hindered by the limited availability of primary cells, especially when specialized T cell subpopulations rather than bulk lymphocytes are the subject of the study.

To overcome some of these shortcomings, we optimized a workflow that allows the functional analysis of proteins of interest directly in primary human T lymphocytes through CRISPR-Cas9 gene-editing followed by T cell cloning and T7 endonuclease I cleavage assay. This method allows the interrogation of protein functions in T lymphocytes regardless of their intracellular location and irrespective of the availability of commercial antibodies for protein detection.

## Materials and methods

### Primary human CD4^+^ T cell isolation and activation

Blood from healthy donors was obtained from the Swiss Blood Donation Center of Basel and Lugano (Switzerland), with informed consent from the Swiss Red Cross and authorization number CE 3428 from the Comitato Etico Canton Ticino. Peripheral blood mononuclear cell (PBMCs) were separated by gradient centrifugation (Ficoll-Paque Plus; GE Healthcare). CD4^+^ T helper lymphocytes were then enriched from PBMCs by positive selection using magnetic microbeads (Miltenyi Biotec), following manufacturer’s instructions. Memory CD4^+^ T cells were further separated by cell sorting using a FACSaria (BD Bioscience), based on the expression of surface markers as follows: CD4^+^CD25^–^CD45RA^–^CCR7^+/–^. For T lymphocyte activation, cells were stimulated for 48 h with plate-bound anti-CD3 (clone TR66, recombinant, made in-house) and anti-CD28 (1 μg, BD Bioscience) antibodies, using NUNC 96-well plates (ThermoFisher), and then re-plated and expanded as needed. T cell culture medium was RPMI-1640 supplemented with 5% human serum, 1% non-essential amino acids, 1%, sodium pyruvate, 1% glutamine and 50 μM β-mercaptoethanol, 1% penicillin and streptomycin (complete medium).

### Considerations for gRNA design and controls

Many online tools are available for the design of gRNAs that achieve high on-target efficiency with relatively low off-target effects [12–14]. We found that the use of more than one gRNA per gene locus can lead to high efficiency of deletion without increasing cell death. However, high editing efficiency depends primarily on the quality of the gRNA, and one single high-quality gRNA can also be as effective. The ideal experimental setup will therefore depend on the specific gene to be deleted and on the quality of the gRNAs. Furthermore, the gene structure should also be taken into account, for instance by avoiding exon-intron boundaries (which may lead to the excision of the intervening intron without significantly affecting protein expression), or alternative splice junctions. In this study, oligonucleotides specific for the gene of interest were designed using a gRNA design online tool (Dharmacon, IDT). These RNA oligonucleotides contain a 20 nt target-specific sequence with a protospacer and a 16 nt sequence complementary to an ATTO-550-labelled tracrRNA (Dharmacon, IDT). Sequences were then manually screened based on their on-target and off-target score (which should be ideally both >70) and their position in the gene body, to give preference to the first exons. Negative controls for this experimental design include mock transfected cells (electroporated without any reagent) or with Cas9 protein only, or transfected using a non-targeting scrambled gRNA or gRNAs against a gene different from the one under consideration and not associated with the phenotype to be observed.

### Transfection with fluorescent oligonucleotides and CRISPR-Cas9 ribonucleoproteins

For transfection optimization, about 7x10^5^ primary memory T cells were transfected with NEON nucleofector (Invitrogen) at different voltages, pulses and widths, using the provided buffer T. A fluorescent siGLO oligonucleotide was used at the concentration of 1–2 μM. For the transfection of CRISPR-Cas9 ribonucleoproteins (RNPs), the selected oligonucleotides (Dharmacon, IDT) were mixed with the fluorescently labelled tracrRNA at a final concentration of 80 μM in 10 μl of nuclease-free duplex buffer (Dharmacon, IDT), followed by annealing by boiling and cool-down, to form the functional gRNA recognized by *Streptococcus pyogenes* Cas9. The annealed gRNA can be aliquoted and stored at -80°C for subsequent use. The RNP complex was instead prepared freshly immediately before transfection by mixing 7.5 μg of recombinant TrueCut Cas9 Protein v.2 (ThermoFisher) with 1.5 μl of the annealed gRNA in a total volume of 3 μl followed by incubation for 20 min at ~25°C. To improve transfection efficiency, Alt-R electroporation enhancer (Dharmacon, IDT) was added to the mix at a final concentration of 1.7 μM. About 7x10^5^ freshly sorted CD4^+^ T cells were resuspended in Neon electroporation buffer T and electroporated using the 10 μl Neon transfection system kit, with one pulse at 2’200 V, width 20 ms, unless otherwise specified. Transfected cells were incubated 24 h in pre-warmed RPMI-1640 medium supplemented with 5% human serum, 1% non-essential amino acids, 1%, sodium pyruvate, 1% glutamine and 50 μM β-mercaptoethanol (complete medium without antibiotics). Transfection efficiency was determined by measuring the intracellular fluorescence of the ATTO-550-labelled tracrRNA by flow-cytometry 24 h post-transfection, compared to control, mock transfected cells (no RNP). To assess the efficiency of gene targeting at the level of protein or gDNA, T cells were cultured for at least 72 h after transfection. The sequences of the RNA oligonucleotides used for targeting are indicated in Table 1.

**Table 1:** Sequence of gRNA oligonucleotides and PCR primers.

**Gene Name**	**gRNA sequence**
*TRAC*	5`- TGTGCTAGACATGAGGTCTA -3`
*B2M*	5`- AAGTCAACTTCAATGTCGGA -3`
*ZC3H12D*_g1	5`- CCTGGTCAACGACGTGCTGC -3`
*ZC3H12D*_g2	5`- CACTATGGGTCGCAGAGAAC -3`
*TBX21*_g1	5`- ACCTCAACGATATGCAGCCG -3`
*TBX21*_g2	5`- GATTAAACTTGGACCACAAC -3`
*TBX21*_g3	5`- CGTCCACAAACATCCTGTAG -3`
Scrambled Control	5`- GGUUCUUGACUACCGUAAUU -3`
**Name**	**Sequences of PCR primers for knock-out screening**
*TRAC*- T7Endo-FW	5`- GACGCAGGTGTTCTGATTTATAGTT -3`
*TRAC*- T7Endo-Rev	5`- TAGAATGAGGCCTAGAAGAGCAGTA -3`
*ZC3H12D* Short-FW	5`- CTGGTCAACGACGTGCTGCAGGA -3`
*ZC3H12D* Short-Rev	5`- CGCAGAGAACTGGCCAGGGTT -3`
*ZC3H12D*_T7Endo-FW	5`- AGTCTGAGAAACAAGAAACCTGTGT -3`
*ZC3H12D*_T7Endo-RV	5`- GTTAGGGACAGACTCCCAACAAG -3`
*TBX21*_ T7Endo-FW	5`- GCAGCACCGCTACTTCTACC -3`
*TBX21*_T7 T7Endo-Rev	5`- CACACACCAGACATATGATCTCAA -3`

### Primary CD4^+^ T lymphocyte single cell cloning

Primary T cells transfected with RNPs targeting the gene of interest were cloned by limiting dilution as described [15]. Briefly, 24 h after transfection, T cells were seeded in complete medium in 384-well plates at a concentration of 0.5 cells/ well and in the presence of recombinant human IL-2 (500 U/ml, made in-house), 1 μg/ml phytohemagglutinin (Remel) and 5x10^5^ irradiated (45 Gy) allogeneic feeder cells/ml (PBMCs) as previously described [4, 15]. After 14 days, individual clones were picked and transferred into round-bottom 96-well plates and further expanded in the presence of IL-2.

### Analysis of gene deletion efficiency

Genomic DNA from primary human T cells and single T cell clones was isolated using the QIAamp DNA Micro Kit (Qiagen) (1x10^4^ to more that 1x10^5^ cells can be used, depending on the ability of each individual clone to expand). To screen for the presence of indels/mutations in the genomic region of interest, a T7 endonuclease I cleavage assay was performed as previously described [4], using PCR primers flanking the targeted gDNA region, and generating a PCR amplicon of ~1000 bp (“long” PCR). Briefly, the PCR reaction was performed using the high fidelity KOD Hot Start DNA polymerase (Novagen) and 100 ng of gDNA template in 30 μl of total volume. Because the T7 endonuclease I enzyme recognizes and cleaves mismatched heteroduplex DNA, 8.5 μl of PCR product were denatured (95°C, 5 min) after addition of 1 μl of 10x NEB Buffer 2 (New England Biolabs), and re-annealed by gradual cooling to produce potential heteroduplexes of wild-type and mutated DNA strands. Then, 5 units of T7 endonuclease I enzyme (New England Biolabs) were added directly to the annealed PCR product and incubated at 37°C for 15 min. As a control, a parallel reaction was performed without the addition of T7 endonuclease I. The reaction was stopped by adding 1.6 μl of 6x gel loading dye (New England Biolabs) and the resulting products resolved on a 1% agarose gel to display cleavage at heteroduplex mismatch sites. When using two RNPs targeting two different regions of the same gene (thereby leading to a deletion of the intervening sequence in at least a proportion of the cells), an independent confirmation of the presence of deletions could be obtained also by designing primers complementary to the region to be deleted (“short” PCR). Upon efficient deletion of this region, or mutation of the primers’ target sequences, the primers would be therefore incapable of generating any PCR product. PCR primers used for these assays are indicated in Table 1. Specific for the *TRAC* and *B2M* genes, efficiency of deletion was also measured by surface staining using anti-human CD3-APC-Cy7 and anti-human β2-microglobulin-APC antibodies (Biolegend).

### Quantification of T7 endonuclease I cleavage assay

Images of the agarose gels displaying the results of the T7 endonuclease I cleavage assay were taken using an INgenius3 instrument and loaded in ImageJ [16]. Using the “Gel analysis tool” each gel line was selected and profile plots were generated, which displayed the intensity of pixels within the selected area along a line. Background subtraction was performed by drawing a baseline on the bottom of each density profile peak, excluding the underlying area from the measurement. The area below each peak was measured using the “Wand tool” and values were used to calculate the percentage of cleavage efficiency using the following formula: % gene modification = 100 x (1- (1 –fraction cleaved)^1/2^), as previously described [17].

### Intracellular cytokine staining and measurement of cell death

Memory CD4^+^ T cells were stimulated for 5 h with PMA (phorbol 12- myristate 13-acetate, 200 nM) and ionomycin (1 μg/ml). For the last 2.5 h of stimulation, brefeldin A (10 μg/ml) was added to the cells. After fixation (paraformaldehyde, 4%) and permeabilization [0.5% BSA and saponin in Dulbecco’s phosphate-buffered saline (DPBS)], the staining was performed with the conjugated antibody anti-IFN-γ-APC/Cy7 (Biolegend) following manufacturer’s instructions. All samples were acquired on a Fortessa Flow Cytometer (BD Bioscience) and data was analyzed with FlowJo Software. To determine the extent of cell survival and cell death, T cells were stained with a live/dead dye (LIVE/DEAD Fixable Aqua Dead Cell Stain Kit; Life Technologies).

## Results and discussion

### CRISPR/Cas9-mediated deletion of surface TCRα expression in primary human CD4^+^ T cells

**Fig 1:**
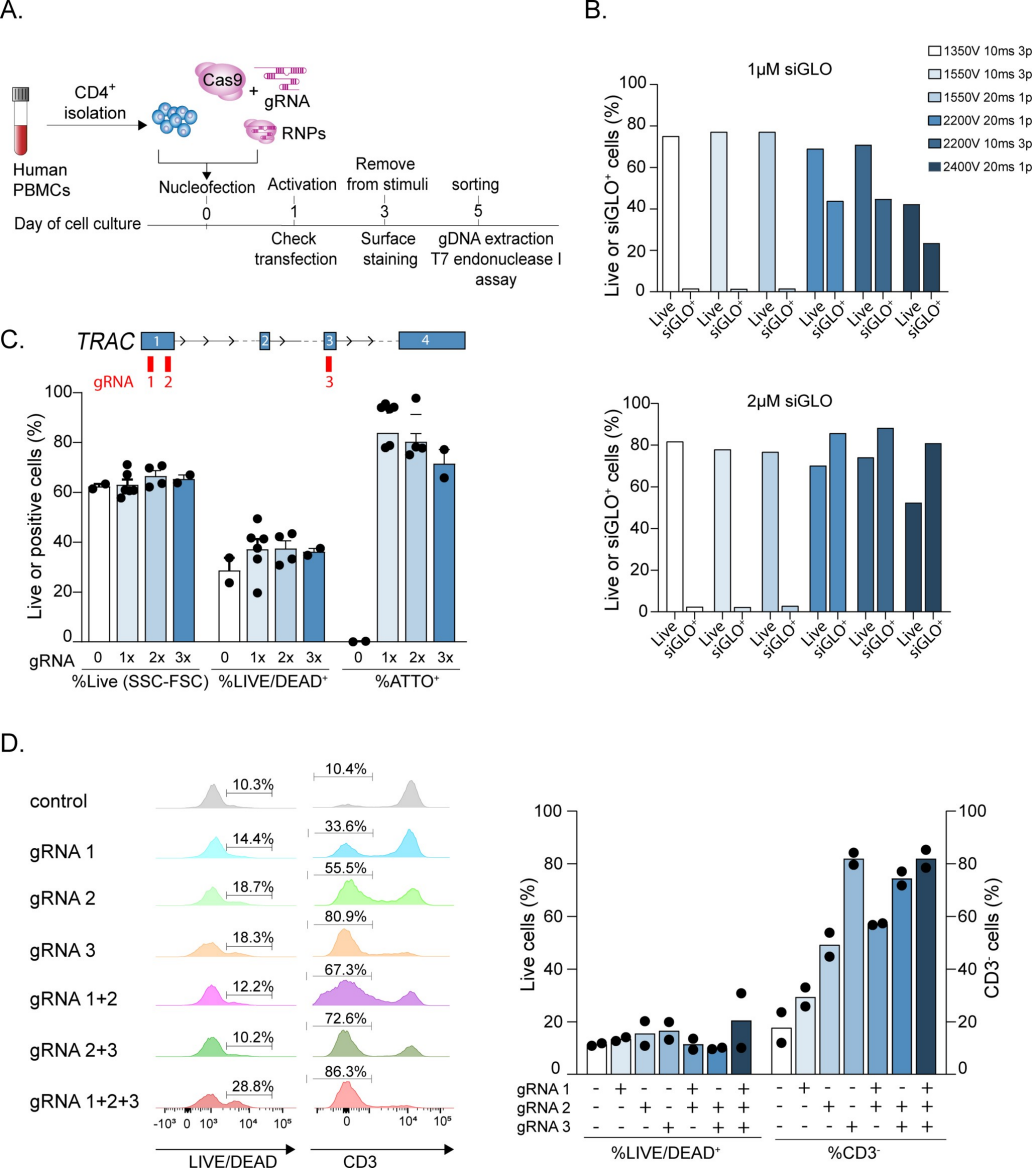
Optimization of transfection conditions for gene editing in primary human T lymphocytes. **(A)** Overview of the experimental workflow, including T cell isolation from the peripheral blood, RNP transfection, cell activation, sorting and downstream analyses. **(B)** Primary human memory T lymphocytes were transfected with 1–2 μM of fluorescent siGLO oligonucleotide using the indicated Neon settings. 24 h after transfection cell viability and efficiency of siGLO incorporation were determined by flow cytometry (n = 1). **(C)** Resting primary memory human T lymphocytes were transfected with Cas9 RNPs including one, two or three different sgRNAs against the same gene (*TRAC*) and the optimized Neon settings (2200V 20ms 1 pulse). The position of the gRNAs relative to the *TRAC* gene is indicated in the scheme on top. Blue numbered rectangles indicate exons. 24 h after transfection the percentage of cell death was assessed by live-dead staining. The percentage of alive cells as assessed by gating on the side and forward scatter parameters (SSC, FSC) is also indicated. The efficiency of transfection was measured based on the incorporation of a fluorescent tracrRNA (ATTO^+^) (n = 2–6). **(D)** Same as (C), except that 24 h after transfection cells were activated with plate-bound anti-CD3 and anti-CD28 antibodies. 3 days after activation, cell viability was assessed by live-dead staining and the efficiency of TCR deletion by surface staining. Left: flow cytometry analysis of cell death and TCR deletion efficiency in a representative donor. Right: results of n = 2 independent experiments.

As a representative example of deletion of a surface protein on primary T lymphocytes, we selected the *TRAC* gene, encoding the TCRα chain. The general overview of the experimental setup is shown in **Fig 1A** and includes the separation of primary human CD4^+^ T lymphocytes from peripheral blood, the transfection with RNPs composed of recombinant Cas9 and the *TRAC* gene-targeting gRNAs, the stimulation, sorting and analysis of the deleted cells. The desired T cell subsets can be separated from PBMCs based on the expression of surface markers as described [4, 18]. For the experiments described here, memory CD4^+^ T lymphocytes were used, but similar results were obtained also for naïve T cells.

First, electroporation conditions for freshly isolated, resting primary T cells were optimized to achieve high transfection efficiency and limited cell death. Using a fluorescent oligonucleotide (siGLO) to gauge transfection efficiency and a panel of Neon settings, we found that several conditions led to high cell viability, but low efficiency of transfection (e.g. 1350V 10ms 3 pulses), while others showed somewhat improved efficiency but higher mortality (e.g. 2400V 20ms 1 pulse). Finally, intermediate conditions (e.g. 2200V 20ms 1 pulse) provided a good balance between moderate cell death and high efficiency (**Fig 1B**). Using the latter conditions, we found that transfecting one, two or three gRNAs against the same gene (*TRAC*) did not alter significantly neither cell survival nor the efficiency of transfection, measured by the percentage of ATTO^+^ cells (**Fig 1C**), indicating that the transfection of multiple gRNAs does not significantly affect the system. Next, we investigated the effect on protein expression of transfecting one, two or three different sgRNAs against the same gene, alone or in combination. We found that the efficiency of deletion was largely dependent on the sequence of the individual gRNAs (**Fig 1D**). Indeed, combining different gRNAs led to an efficiency similar to the most effective gRNA in the mix, without altering the level of cell death. In the specific example shown in **Fig 1D**, the three gRNAs showed increasing efficiency (gRNA 1 lowest, gRNA 3 highest), and gRNA 3 provided similar efficiency whether it was used alone or in combination with other gRNAs. Overall, we optimized conditions that allowed effective gene editing in T lymphocytes with limited cell death.

**Fig 2:**
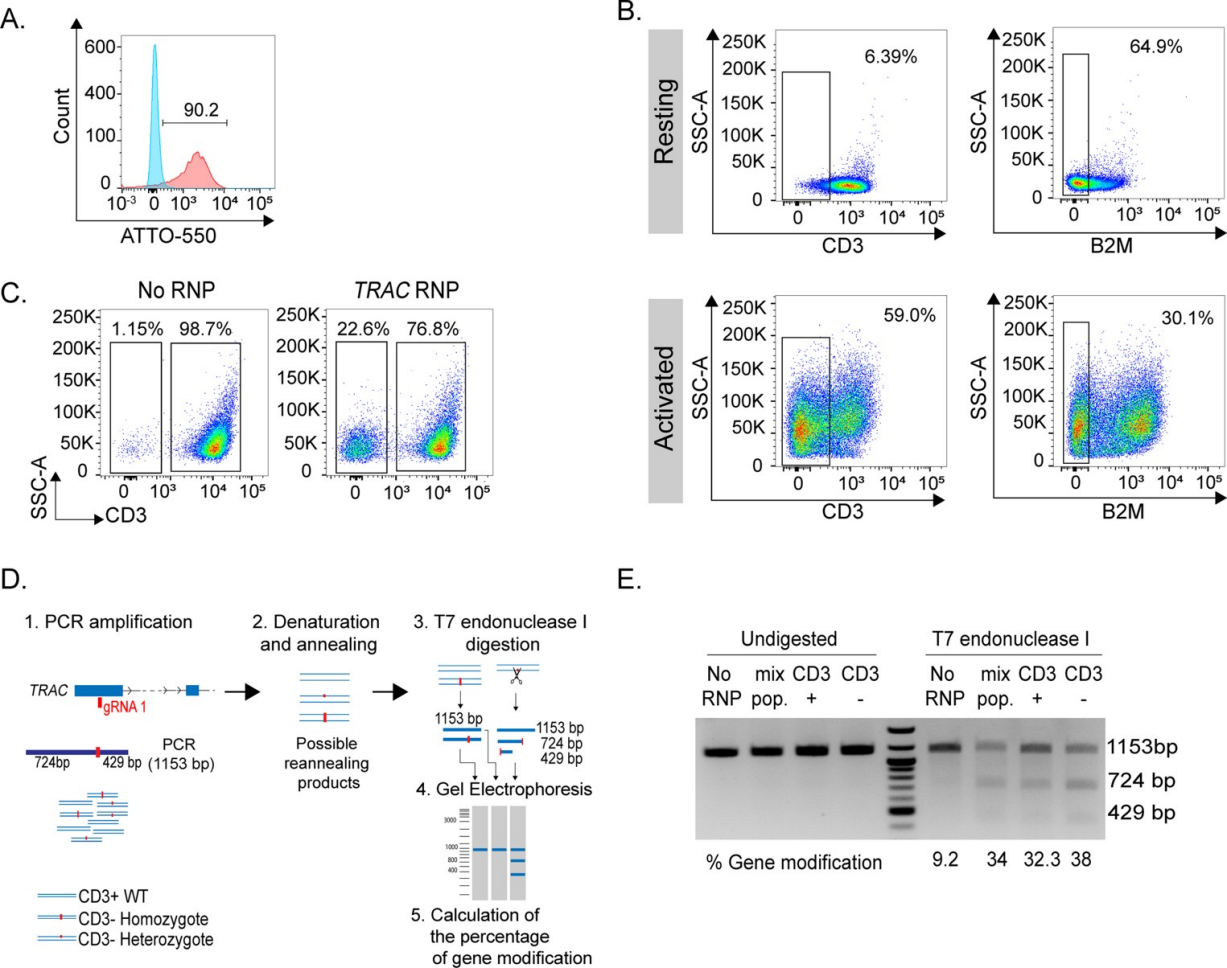
Deletion of surface proteins in primary human T lymphocytes. **(A)** Example of efficiency of RNP transfection in resting memory T lymphocytes, determined by measuring the fluorescence of the ATTO-550-labelled tracRNA (red). Blue: no RNP control (at least n = 2). **(B)** Surface protein ablation upon RNP transfection in resting and activated primary human T lymphocytes (n = 1). **(C)** Efficient surface ablation of the TCR complex in resting T cells transfected with *TRAC* gene-targeting RNPs and activated (at least n = 2). **(D)** Overview of the T7 endonuclease I cleavage assay, including the location of the *TRAC*-targeting gRNA, the PCR amplification, leading to mixed DNA populations, denaturing and reannealing, digestion with T7 endonuclease I and quantification. **(E)** Result of a representative T7 endonuclease I cleavage assay performed on cells as in **(C)**, either unsorted or sorted based on CD3 expression (n = 2).

Next, we assessed the efficiency of deletion in resting and activated T lymphocytes. The efficiency of transfection was determined in all cases by measuring the fluorescence derived from the ATTO-550-tracrRNA (**Fig 2A**). In resting cells, three days after transfection there was only minimal decrease in TCR surface expression, evaluated by flow cytometry using an anti-CD3 antibody (**Fig 2B**). However, this depended primarily on the stability of the protein on the surface. Indeed, a decrease in the surface expression of a less stable protein, namely β2-microglobulin [5], could be observed in >64.9% of the cells even without activation (**Fig 2B**). Editing of the same genes in activated cells led to an almost 60% reduction of TCR expression upon *TRAC* gene editing, and about 30% reduction for β2-microglobulin. The relative stability of the surface TCR complex provided a window of opportunity for T cell activation using plate-bound anti-CD3 and anti-CD28 antibodies even in cells that successfully underwent gene deletion while transfected at the resting state. Three days after transfection and two days after activation, flow cytometry analysis showed that 22.6% of transfected CD4^+^ primary human T cells lost surface TCR expression, consistent with successful gene targeting (**Fig 2C**). The remaining 76.8% of cells maintained TCR expression at a level that was comparable to control (no RNP) transfected cells (**Fig 2C**). Overall, we found that effective deletion by CRISPR-Cas9 is possible in both resting and activated T lymphocytes, although the overall efficiency depends on the many factors, including the quality of the targeting sgRNA and the stability of the protein of interest.

RNP transfection leads to a mixed population in which mutations and indels can potentially be present on one (heterozygote), two (homozygote) or no (wild-type) alleles (**Fig 2D**). We therefore evaluated the efficiency of TCRα targeting at the level of gDNA by T7 endonuclease I cleavage assay. This method provides a semi-quantitative readout of the percentage of alleles mutated within a mixed cell population [17], and an overview of the method is shown in **Fig 2D**. Briefly, PCR oligonucleotides were designed to amplify a 1153 bp genomic region flanking the location of the gRNA. Denaturation and re-annealing of the PCR product leads to the formation of heteroduplexes between wild-type and mutant amplicons, if mutations or indels are present in at least a portion of the cell population. Digestion of these heteroduplexes with the T7 endonuclease I enzyme leads to the formation of cleavage products that can be resolved on a gel and quantified. The frequency of mutations can be then estimated by comparing control (no T7 endonuclease I) and experimental samples. To assess the frequency of genomic modifications (mutations, indels) in the transfected T cell populations shown in **Fig 2C**, CD3^+^ and CD3^–^ cells were separated by sorting and the gDNA was extracted and used for T7 endonuclease I cleavage assay. A PCR product of the expected size was observed in all samples, including the no RNP control, the mixed, unsorted population, as well as the sorted CD3^+^ and CD3^–^ populations (**Fig 2E**). Digestion with the T7 endonuclease I enzyme gave rise to two smaller bands of 724 bp and 429 bp, most prominently in the CD3^–^ population, but also in the mixed population and in the sorted CD3^+^ cells. The no RNP control showed no meaningful digestion. The presence of cleavage products in CD3^+^ cells despite levels of TCR expression comparable to those of control cells was likely due to heterozygous mutations (therefore leaving one perfectly functional allele) and/ or to small mutations and indels that ultimately did not affect TCRα protein expression. On the other hand, the remaining uncleaved product that was observed in CD3^–^ cells was likely due to the re-annealing of sequences containing the exact same mutations (**Fig 2E**). The level of purity of the populations after sorting should also be taken into consideration. Nevertheless, the calculated percentage of gene modification was higher in sorted CD3^–^ cells, lower in CD3^+^ cells and intermediate in the mixed population, reflecting the observed phenotype. Taken together, these results show that the transfection with RNPs allows the efficient deletion of proteins expressed on the surface of primary human T lymphocytes, both resting and activated, and that the T7 endonuclease I cleavage assay is a relatively quick and cost-effective method to estimate RNP-induced mismatches in T cells.

### Considerations for the RNP-mediated deletion of intracellular factors and T cell cloning

Performing gene targeting in primary human T cells using RNP electroporation leads to a diverse and heterogeneous cell population containing variable proportions of wild-type and mutated alleles of the gene of interest, as well as different types of mutations. Variables influencing the fraction of deleted cells include the electroporation conditions, the T cell subset that needs to be transfected, whether cells are already activated or resting and the quality of the gRNAs available for the gene of interest, which can be designed using many different tools. The gRNAs used in this study were designed using the Integrated DNA Technologies online tool (www.idtdna.com/CRISPR-Cas9). The gRNA design has to be tailored to each target gene depending on the gene structure. Generally, it is preferable to target the first 40% of the coding sequence, to increase the probability of obtaining a loss-of-function mutation after non-homologous-end-joining (NHEJ) [19]. However, to target particular sites of the protein (e.g. catalytic or transmembrane domains) or a particular isoform it may be required to select appropriate gRNAs that target those specific regions. The use of one single gRNA can induce random indels and mutations that can be sufficient to result in the loss of protein expression (**Fig 1**).

**Fig 3:**
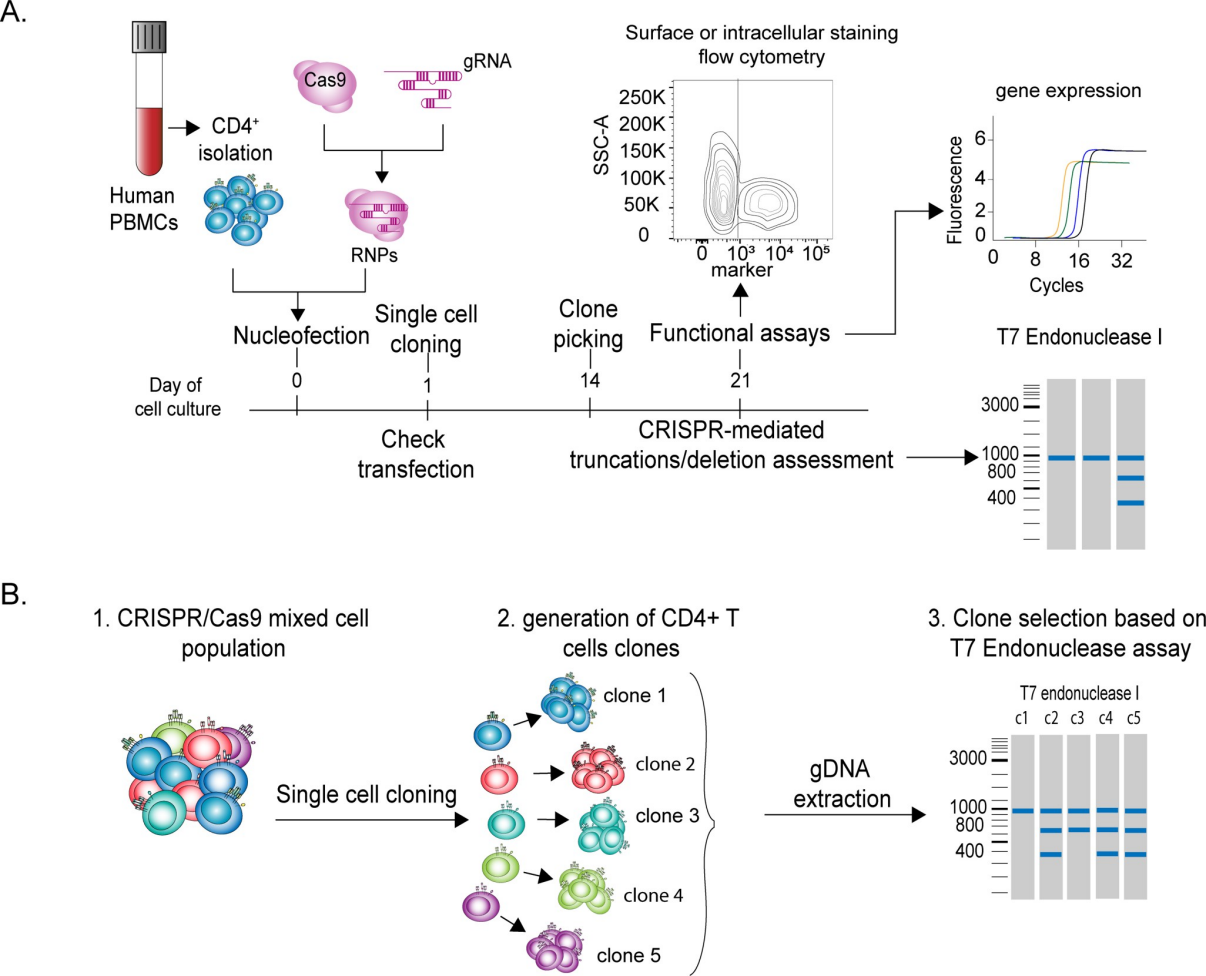
Deletion of intracellular proteins by gene editing, coupled to T cell cloning. **(A)** Overview of the experimental workflow, including T cell isolation from the peripheral blood, RNP transfection, single cell cloning and expansion, gDNA screening and functional analyses. **(B)** Schematic representation of the single-cell cloning procedure followed by T7 endonuclease I cleavage assay.

Selecting T lymphocytes that underwent successful gene editing might be challenging, especially when it comes to the study of intracellular proteins. Thanks to cell sorting, the selection of live cells that effectively deleted the gene of interest can be relatively straightforward for surface proteins. Instead, in the case of intracellular proteins or non-coding RNAs the selection of modified cells may become extremely challenging. For instance, availability of an antibody for intracellular staining may allow the separation of fixed and permeabilized cells, which may be useful for some specific downstream analyses, but it does not allow the culture of live cells and thereby the study of T cell responses. Another possibility to select live cells that effectively deleted the gene of interest is by exploiting homology direct DNA repair (HDR) mechanisms. HDR enables precise genome editing through the delivery of an HDR template (single- or double-stranded DNA) together with RNPs targeting the gene of interest. Although technically challenging, HDR allows the engineering of genomic changes such as base substitutions, sequence insertions or deletions at defined genomic regions, including the introduction of restriction sites or entire reporter genes [2, 20, 21]. However, this procedure is more time consuming and much less efficient compared to the regular RNP transfection of T lymphocytes and it requires extensive cell manipulation. The selection of live cells lacking a gene encoding an intracellular protein or a non-coding RNA remains therefore technically challenging. One possibility in this direction is to derive single-cell clones from transfected T cells, and to study their phenotype after selection of effectively deleted clones by T7 endonuclease I cleavage assay. The workflow of such experimental setup is shown in **Fig 3A**. Briefly, after cell separation and transfection, individual T cells are seeded in 384-well plates by limiting dilution, and stimulated with irradiated allogeneic PBMCs and phytohemagglutinin, in the presence of IL-2 to sustain proliferation [4, 15, 22]. Individual clones are picked after ~14 days, transferred in 96-well plates with U-bottom and further expanded prior to gDNA extraction and functional analyses. Individual clones can be then selected based on the percentage of gene modification determined by T7 endonuclease I cleavage assay (**Fig 3B**). A drawback of the cloning procedure is clearly linked to the function of the gene that has to be deleted. For instance, if the gene of interest is required for T cell proliferation or survival, no deleted clone will be obtained. Moreover, long-term T cell cultures are characterized by extensive proliferation that might affect the phenotype under analysis, for example the amount of some specific cytokines that the cells may become able to produce [4]. Notwithstanding these considerations, coupling T cell cloning to T7 endonuclease I digestion is an effective approach to select cells in which a successful event of gene editing occurred and to obtain homogeneous cell populations after deletion.

### Deletion of an intracellular factor in primary human T lymphocytes and selection of deleted clones

**Fig 4:**
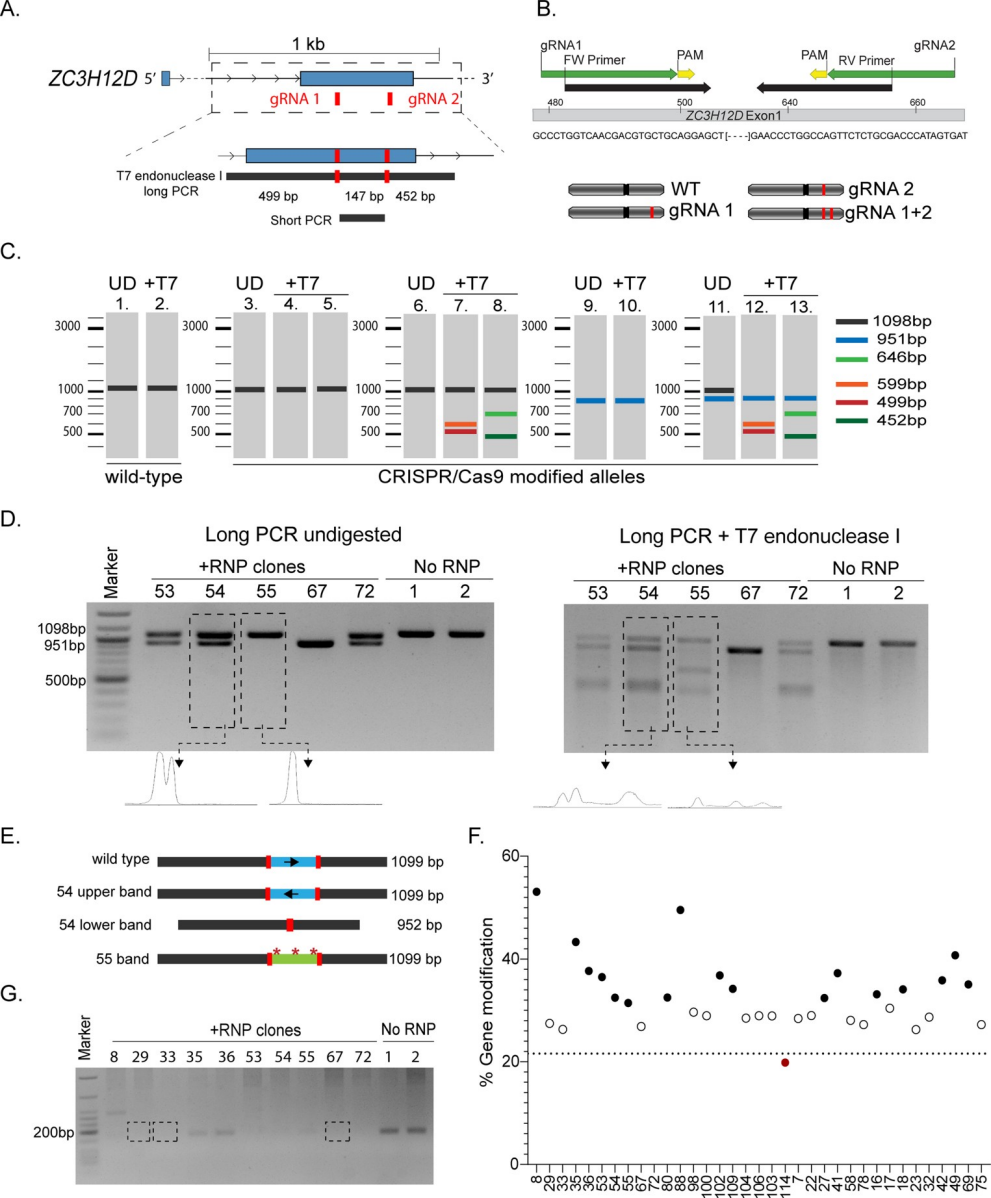
Deletion of the *ZC3H12D* gene in primary human T lymphocytes. **(A)** Schematic representation of the *ZC3H12D* gene with indicated the location of gRNAs and PCR products. **(B)** Top: location of the “short PCR” primers compared to the location of the gRNAs and the *ZC3H12D* genomic sequence. Bottom: RNP transfection may lead to alleles that are unmodified (WT), modified only at the location of one gRNA (gRNA 1 or 2), or modified at both locations (gRNA 1+2), leading to the possible alleles shown. In each cell, all combinations of any of these two alleles are also possible (red: gene modification). **(C)** Schematic representation of all the possible outcomes of the T7 endonuclease I cleavage assay performed on different combination of alleles as in **(B)** UD = undigested. **(D)** After transfection with RBPs targeting the *ZC3H12D* gene and cell cloning, individual T cell clones were expanded and the gDNA analyzed for the presence of indels/ mutations by T7 endonuclease I cleavage assay. The panels show the PCR product before (left) or after (right) digestion with the T7 endonuclease I enzyme. The density plots show representative quantifications (Representative clones from two independent donors) No RNP = mock transected control clones. **(E)** Schematic representation of the Sanger sequencing results of the PCR bands for clones 54 and 55 shown in panel **(D)**. **(F)** Percentage of gene modification of clones analyzed as in **(D)** (n = 35 clones). **(G)** Short PCR to assess the presence of DNA deletions between the two gRNAs (n = 12 clones from two independent donors).

As an example of intracellular factor to be deleted, we selected the *ZC3H12D* gene, encoding for Regnase-4, and RNase enzyme able to restrain inflammatory cytokine expression in human T lymphocytes [4, 23]. We selected two gRNAs, both of them targeting exon 1 of *ZC3H12D*, separated by 147 bp (**Fig 4A**). The clone selection strategy involved a PCR across the region targeted by both gRNAs (long PCR), followed by T7 endonuclease I digestion. We also designed a “short PCR” (see below), with primers overlapping the sequences of the gRNAs (**Fig 4B**). Because cells have two alleles for this gene, all combinations of mutations/indels are in principle possible and can occur in homo- or heterozygosis (**Fig 4B**). The expected T7 endonuclease I digestion patterns are therefore complicated by the simultaneous use of two gRNAs that can have different outcomes on the two alleles for the same gene and are summarized schematically in **Fig 4C**. First, the expected size of the PCR product for a wild-type allele *ZC3H12D* is 1098 bp, and T7 endonuclease I digestion would result in no cleavage product (**Fig 4C**, lanes 1 and 2). On the other hand, if either gRNA 1 or 2 lead to mutations or very small indels in their target sites that are perfectly identical in both *ZC3H12D* alleles and in all cells (an unlikely scenario), the assay would also result in a single band similar to the wild-type (**Fig 4C**, lanes 3, 4 and 5). More frequently though, a mixture of wild-type alleles and alleles modified by either gRNA 1 or 2 will be present in the population, and this will give rise to a combination of uncleaved and cleaved products ranging from 455 bp to 643 bp (**Fig 4C**, lanes 7 and 8). Finally, if both gRNA 1 and 2 lead to the deletion of the intervening DNA region between the gRNAs, a 951 bp amplicon should be expected (**Fig 4C**, lane 9). If such deletion occurs on both alleles, then the digestion with T7 endonuclease I enzyme does not lead to any further cleavage (**Fig 4C**, lane 10). Finally, a combination of deletions and mutations in the different alleles will give rise to a more complex pattern of digestion (**Fig 4C**, lanes 12 and 13).

After RNP assembly and transfection of freshly isolated CD4^+^ memory T cells, viability and electroporation efficiency were measured. In this particular experiment, the electroporation efficiency was >97%, while the viability was >70%. T cells were then cloned by limiting dilution at 0.5 cells per well. We then expanded 116 clones for the *ZC3H12D* RNP transfected samples and 60 clones for the control sample. The cloning efficiency was calculated as the number of viable clones isolated after 14 days of culture compared to the number of cells initially seeded and was ~11–15%. The cloning efficiency can be affected by the selected sub-population, since for example memory T lymphocytes include also more differentiated effector memory cells, which could be more resilient to undergo the extensive proliferation required during the cloning procedure. The cloning efficiency is also an important parameter that might provide initial functional indications about the gene being targeted. For example, the targeting of specific genes could impair the cloning procedure, for instance in the case of genes crucial for T cell proliferation or survival. Once generated and expanded, individual T cell clones can be used to extract gDNA and for functional analyses and/ or RNA extraction. Although the cell number per clone can be extremely variable, up to 1x10^6^ cells can be obtained. For downstream analyses, we selected 34 clones obtained from cells transfected with RNPs against the *ZC3H12D* gene, and 15 control clones. Using the long PCR described above, we observed that most of the clones displayed modifications at the *ZC3H12D* locus, although at different extent and with different patterns (five representative clones are shown in **Fig 4D**). For example, clones 53, 54 and 72 presented two PCR bands prior to T7 endonuclease I digestion. Upon enzymatic cleavage, a ~500 bp band became apparent. Such pattern is consistent with one allele carrying either mutations or small indels at the target site of the gRNA 1, and the other allele containing a large deletion between the two targeted sites (similar to lanes 11 and 13 in **Fig 4C**). Sanger sequencing of the two PCR bands shown in **Fig 4D** for clone 54 provided valuable information about the mutations that occurred in this clone. Consistent with the results of the T7 endonuclease I digestion, we found that the lower, smaller band for clone 54 contained indeed a deletion of 147 bp, which led to the elimination of the entire genomic region in between the two gRNAs (schematic representation of sequencing results in **Fig 4E**). Sequencing of the larger, top band of clone 54 revealed instead that in this case the intervening region between the two gRNAs was excited and then re-inserted in the opposite orientation, leading to an inversion that effectively disrupted the gene but did not alter the size of the band compared to the wild-type (**Fig 4E**).

Analysis of clone 55 revealed instead a different pattern, with the generation of a ~650 bp band in the cleaved product that is consistent with a mutation introduced by gRNA 2 in one allele, while the second allele remained unmodified or contained only point mutations. Sequencing of the PCR band from clone 55 revealed the presence of multiple mutations that started in close proximity of the two gRNAs, showing that while overall this band corresponds to the size of the wild-type, it is in fact mutated (**Fig 4E**). Finally, clone 67 showed instead the deletion of the DNA region between the two target sites, on both alleles. Because the two alleles contained most likely identical deletions, no cleavage product was observed after T7 endonuclease I digestion (similar to lane 10 in **Fig 4C**).

Next, we calculated a quantitative estimate of the percentage of gene modification in 34 modified clones and 10 control clones, as previously described [17]. Briefly, we generated profile plots for each PCR gel lane, with or without digestion with T7 endonuclease I (examples of the profile plots are shown in **Fig 4D**). The area below each peak was quantified and used to estimate the “fraction cleaved” for each T cell clone analyzed (fraction cleaved = PCR digested/ (PCR undigested + PCR digested)) and subsequently used to calculate the percentage of gene modification [17]. The mean percentage of gene modification obtained from control (no RNP) samples was used to set a threshold (21.7%) below which we considered that no RNP-mediated modification occurred. We found that the majority of modified, experimental clones displayed a percentage of gene modification ranging from ~30 to 53% (**Fig 4F**). This variability might be related to the sensitivity of this assay that can be affected at all stages, from the efficiency of PCR amplification, to the heteroduplex formation and T7 endonuclease I digestion. Moreover, as for other agarose gel quantification assays, the estimated percentage could be greatly affected by the quality of the gel run and imagining, which have to be standardized as much as possible. Overall, the quantification revealed that most selected clones contained a modified *ZC3H12D* locus. Clone 114 did not show any cleavage pattern upon T7 endonuclease I digestion, resulted in a percentage of gene modification comparable to the control clones (**Fig 4F**, red dot) and was therefore considered as unmodified. It is important to notice however, that in T cell clones like clone 67, presenting an identical deletion on both alleles that cannot be digested by the T7 endonuclease I enzyme, the percentages of gene modification were close to the one observed for the wild-type clones (**Fig 4F**, white dots), suggesting that careful visual inspection of the PCR and digestion patterns for each clone remains crucial.

As an independent confirmation of the potential deletion between the two gRNAs, we also designed a “short PCR” (schematic in **Fig 4A**) that can give rise to a product only if the genomic region between the two gRNAs is still intact. The absence of a PCR product from clone 67 and similar clones (e.g. 29, 33) confirmed the absence of the DNA region in between the two gRNAs (**Fig 4G**). Concordant with the sequencing results, the “short PCR” of clone 54 and 55 showed no or very inefficient amplification due to presence of mutations already within the sites of annealing of the PCR primers, which overlap with the gRNAs (**Fig 4B**).

Altogether, we identified T cell clones in which a successful editing of the *ZC3H12D* gene occurred. The deletion of this gene in primary human T lymphocytes also affected their phenotype and their ability to produce inflammatory cytokines, as we recently reported [4].

**Fig 5:**
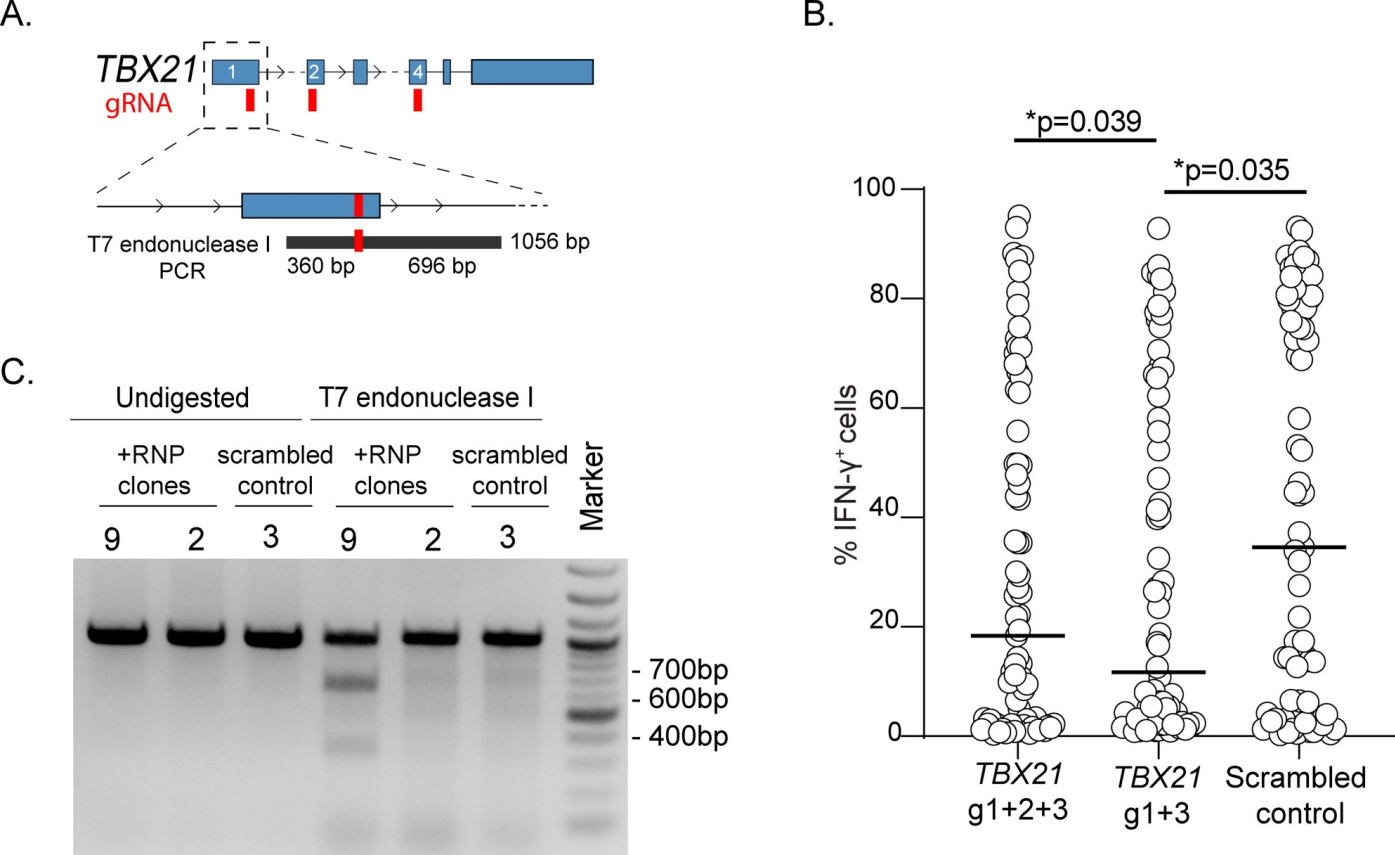
Deletion of the *TBX21* gene in primary human T lymphocytes and functional analysis. **(A)** Schematic representation of the *TBX21* gene with indicated the location of the gRNAs and PCR products. **(B)** After single cell-cloning and expansion, clones transfected with gRNAs against *TBX21* or scrambled control oligonucleotides were stimulated with PMA and ionomycin and IFN-γ expression was measured by intracellular staining. Median ± SD. Each dot represents one individual clone (at least n = 72 clones). Mann-Whitney test. **(C)** Example of T7 endonuclease I digestion of one control clone and one modified clone (representative of n = 21 clones analyzed).

Finally, to assess the general applicability of our method, we applied our workflow to the deletion of the transcription factor T-BET (encoded by the *TBX21* gene) in primary human T lymphocytes and we assessed the functional outcome on IFN-γ expression. First, we designed three different gRNAs within the *TBX21* locus (**Fig 5A**), and we transfected primary memory T lymphocytes with a combination of two or three RNPs containing different gRNAs. After single-cell cloning and expansion, the ability of the cells to produce IFN-γ was measured by intracellular staining and revealed a significant reduction in the fraction of IFN-γ-producing clones in all samples transfected with gRNAs against *TBX21*, as compared to control clones transfected with a scrambled, non-targeting gRNA (**Fig 5B**). Analysis of the gDNA of some of these clones revealed that gene modifications in the *TBX21* locus could be identified as expected (**Fig 5C**).

Overall, our optimized workflow for gene-editing in primary human T lymphocytes is effective and broadly applicable to both surface receptors and intracellular factors.

### General considerations and limitations of the method

Gene-editing using CRISPR-Cas9 vastly expands the tool-box that is at the disposal of researchers interested in studying the mechanisms underlying primary human T cell responses. Because this method is not fully devoid of limitations, a careful consideration of the biological question to be addressed and what is the best experimental approach to tackle such question remains crucial. For instance, an intracellular, abundant, relatively unstable protein that strongly affects T cell phenotype even when only partially downregulated would be probably more easily studied using RNAi-based methods, which would have fever problems of cell selection and extensive T cell cultures. On the other hand, in the case of a stable protein that is highly functional even when its expression is diminished, RNAi would likely not be sufficiently effective and gene-editing at the level of DNA would be required. Similarly, for specific cell phenotypes that may be altered by extensive *in vitro* cultures, or for genes whose deletion may lead to cell cycle arrest or cell death, RNAi may still be the method of choice. Notwithstanding these considerations, having now the possibility to combine different methods to tackle various aspects of the same question will undoubtedly lead us to a better understanding of the regulation of immune responses.

## Conclusions

While the ideal validation of CRISPR-Cas9-mediated knockout remains undoubtedly a protein readout, Western blots or any antibody-based detection system are not always an option due to technical constraints that become especially important when working with primary cells, often available in limited amount. This consideration is especially important when working with intracellular proteins, that cannot be easily detected while maintaining at the same time cell viability, and even more so with non-coding RNAs. While the optimized method described here still relies on the extensive culture of T cells, which may affect the phenotype under analysis and should be therefore carefully evaluated, the T7 endonuclease I cleavage assays and PCR-based screens are relatively easy and rapid methods to evaluate the efficiency of CRISPR-Cas9 gene editing in primary human CD4^+^ T lymphocytes, for both surface and intracellular proteins.

## Supporting information

S1 Raw image (PDF)
