# Supplementary material for: An optimized workflow for CRISPR-Cas9 deletion of surface and intracellular factors in primary human T lymphocytes
Source: PLoS One. 2021 Feb 18;16(2):e0247232. doi: 10.1371/journal.pone.0247232 (PMC7891767; doi:10.1371/journal.pone.0247232)
Supplement: S1 Raw image — (PDF) [file pone.0247232.s001.pdf]

Method used to capture image: UV detection of DNA samples separated on agarose gel containing RedSafe Nucleaic Acid Staining Solution

Image was acquired with Ingenius3

Figure panel generated from thimage: 1F.

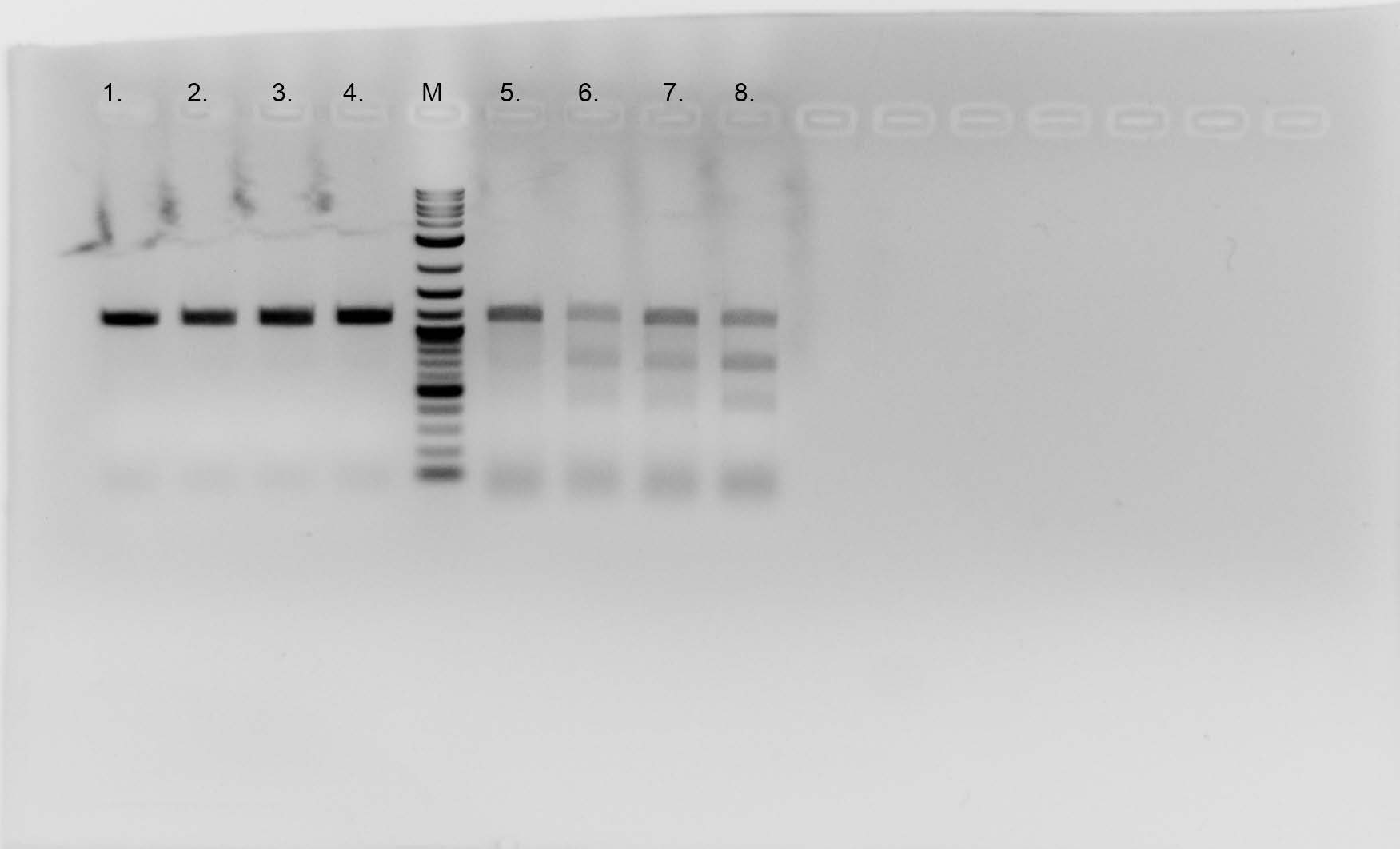

1. No RNP control
2. mix population
3. sorted CD3+
4. sorted CD3-

Undigested PCR

M = marker 1kb NEB

5. No RNP control
6. mix population
7. sorted CD3+
8. sorted CD3-

T7 endonucleas I digested PCR

Method used to capture image: UV detection of DNA samples separated on agarose gel containing RedSafe Nucleaic Acid Staining Solution

Figure panel generated from this image: 3D.

M = marker

Undigested PCR:

+ RNP ZC3H12D:

1. clone nr. 53
2. clone nr. 54
3. clone nr. 55
4. clone nr. 67
5. clone nr. 72

No RNP:

6. control sample 1
7. control sample 2

PCR digested with T7 endonuclease I:

+RNP ZC3H12D:

8. clone nr. 53
9. clone nr.. 54
10. clone nr. 55
11. clone nr. 67
12. clone nr. 72

No RNP:

13. control sample 1
14. control sample 2

marker 1kb Plus DNA ladder (NEB)

Pictures were acquired with Ingenius3

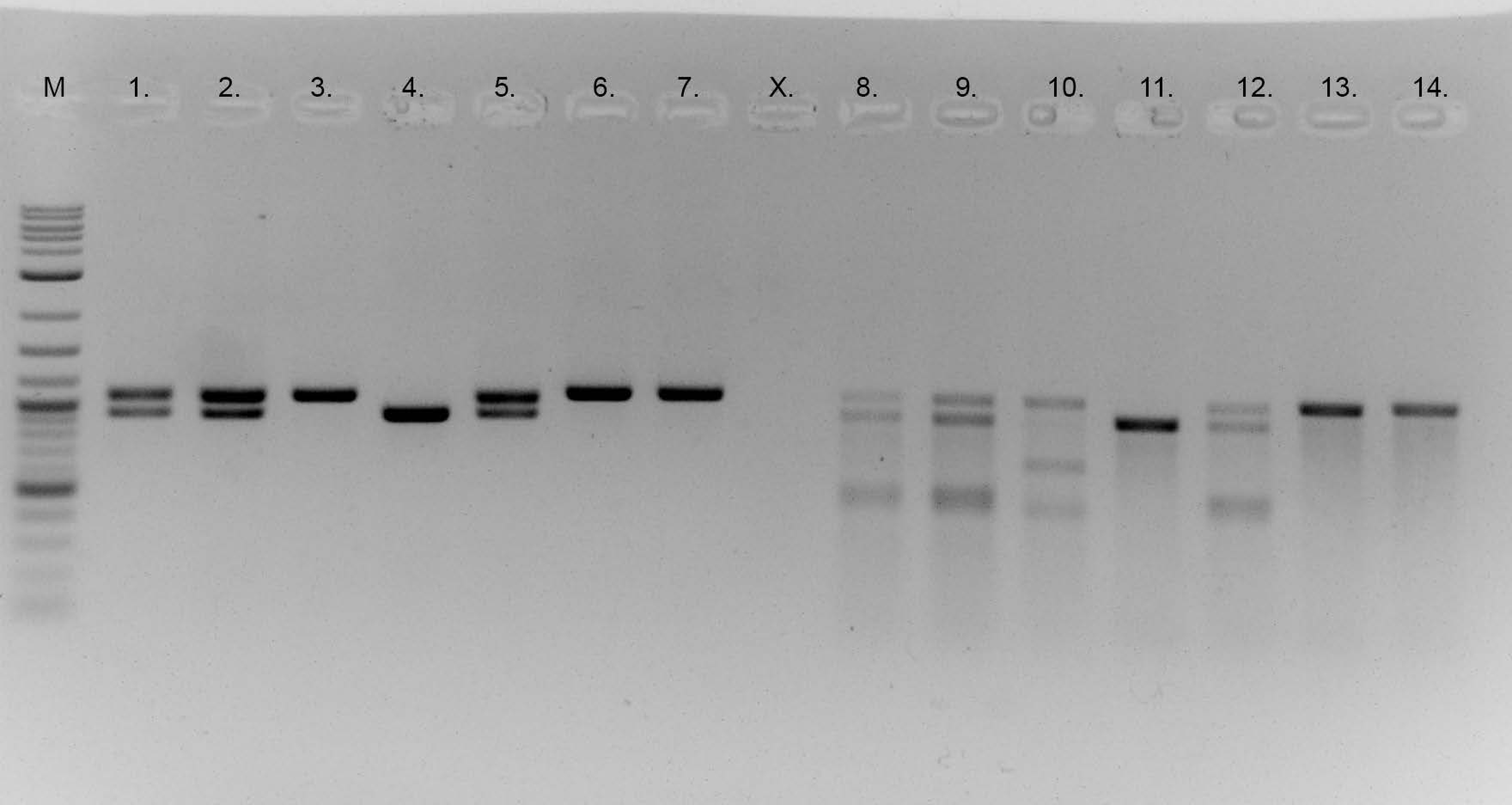

Method used to capture image: UV detection of DNA samples separated on agarose gel containing RedSafe Nucleaic Acid Staining Solution

Figure panel generated from this image: 3F.

M = Marker low molecular weight DNA ladder NEB

1. clone nr. 8
2. clone nr. 29
3. clone nr. 33
4. clone nr. 35
5. clone nr. 36
6. clone nr. 53
7 clone nr. 54
8. clone nr. 55
9 clone nr. 67
10. clone nr. 72

+ RNP

11. control sample nr. 1
12. control sample nr. 2
13. control sample nr. 3
14. control sample nr 4
15. control sample nr. 5

No RNP control

Pictures were acquired with Ingenius3

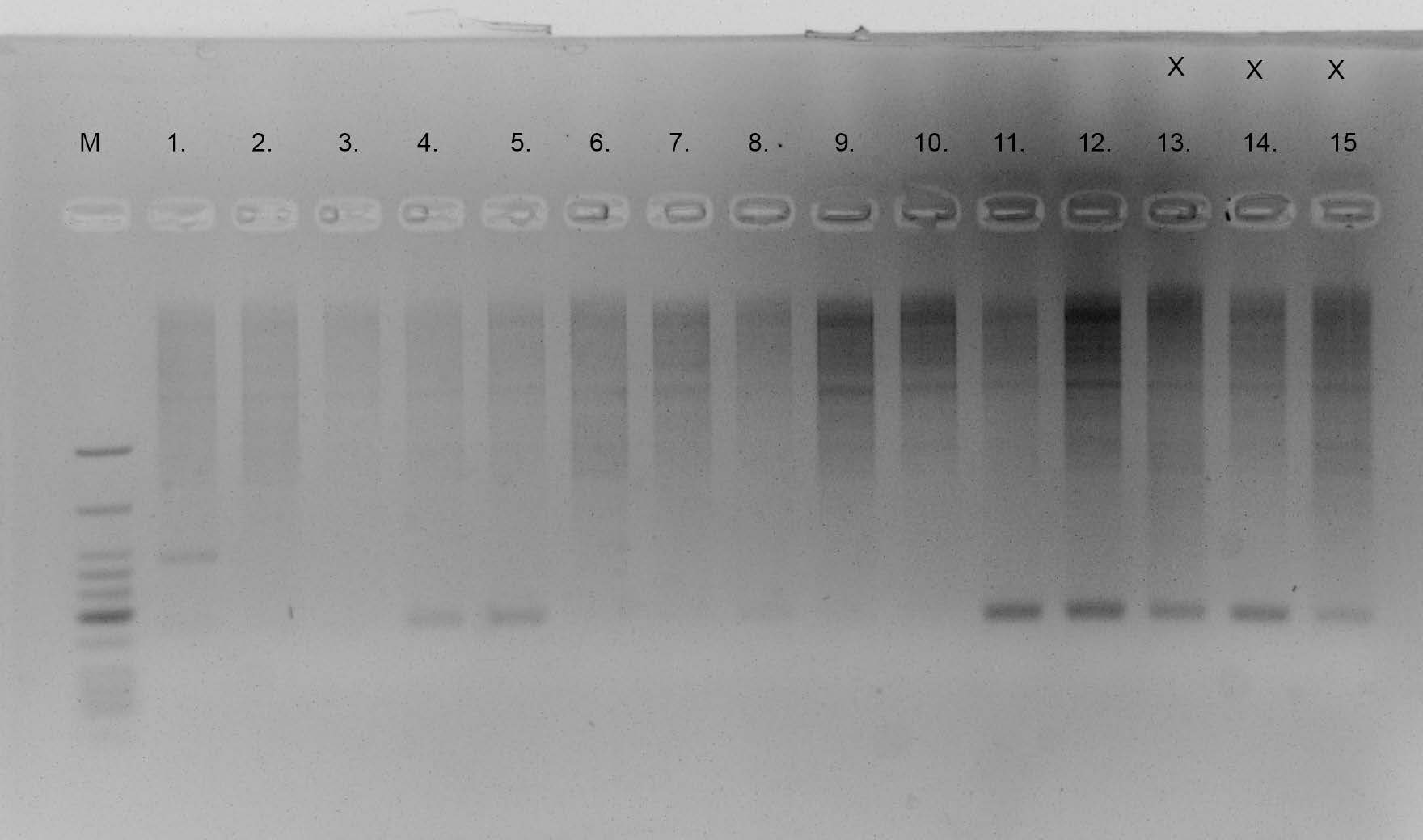

Method used to capture image: UV detection of DNA samples separated on agarose gel containing RedSafe Nucleic Acid Staining Solution

Image was acquired with Ingenius3

Figure panel for image 5C

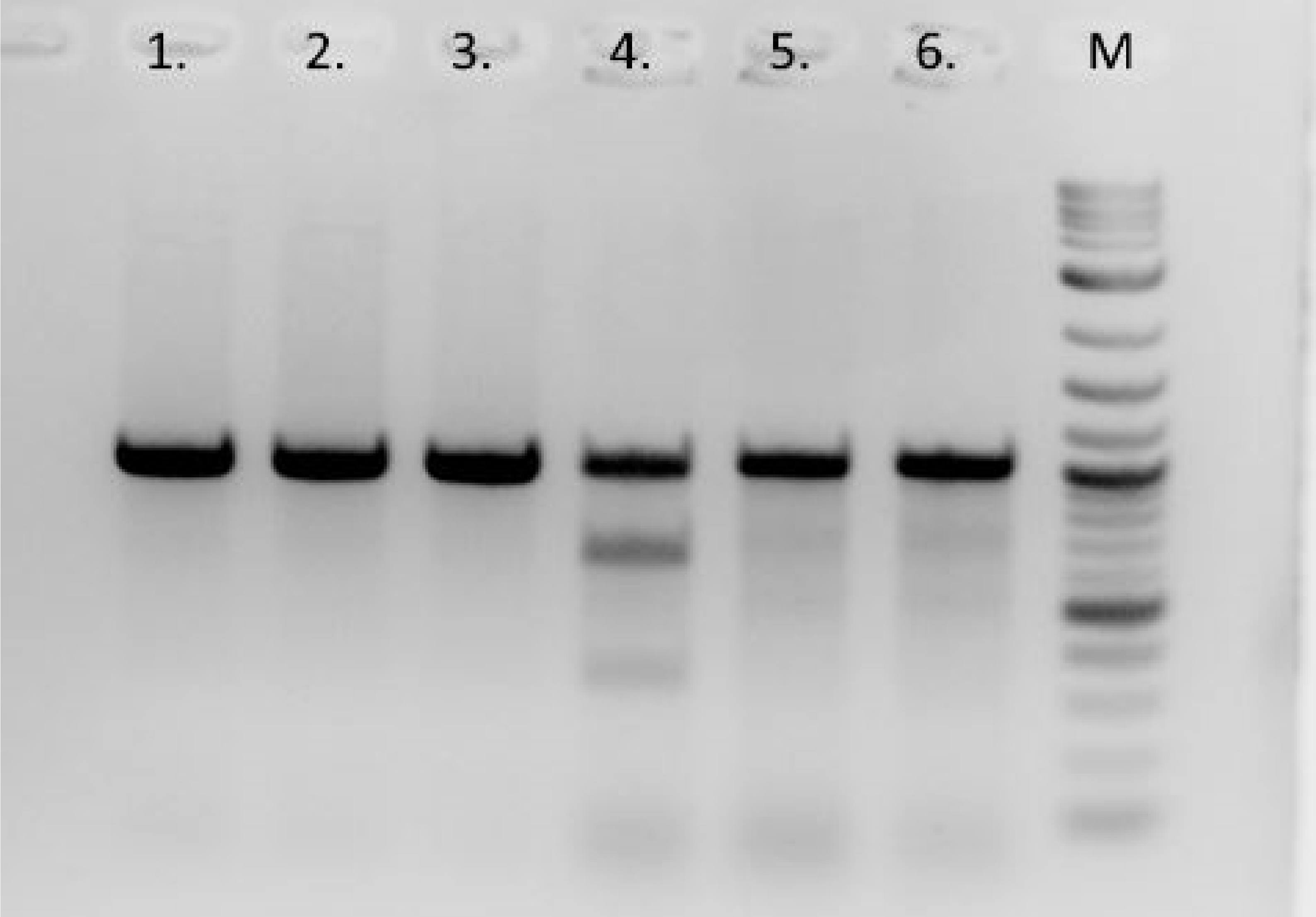

1. clone 9
2. clone 2
3. control 3

No T7 Endonuclease I

4. clone 9
5. clone 2
6. control 3

+T7 Endonuclease I
